# Interaction of Colchicine-Site Ligands With the Blood Cell-Specific Isotype of β-Tubulin—Notable Affinity for Benzimidazoles

**DOI:** 10.3389/fcell.2022.884287

**Published:** 2022-05-31

**Authors:** Felipe Montecinos, Maura Loew, Tak I. Chio, Susan L. Bane, Dan L. Sackett

**Affiliations:** ^1^ Division of Basic and Translational Biophysics, Eunice Kennedy Shriver National Institute of Child Health and Human Development, National Institutes of Health, Bethesda, MD, United States; ^2^ Department of Chemistry, Binghamton University, State University of New York, Binghamton, NY, United States

**Keywords:** tubulin isotypes, beta tubulin, erythrocytes, colchicine, benzimidazoles, repurposing drugs

## Abstract

Tubulin, the main component of microtubules, is an α-β heterodimer that contains one of multiple isotypes of each monomer. Although the isotypes of each monomer are very similar, the beta tubulin isotype found in blood cells is significantly divergent in amino acid sequence compared to other beta tubulins. This isotype, beta class VI, coded by human gene TUBB1, is found in hematologic cells and is recognized as playing a role in platelet biogenesis and function. Tubulin from the erythrocytes of the chicken *Gallus gallus* contains almost exclusively βVI tubulin. This form of tubulin has been reported to differ from brain tubulin in binding of colchicine-site ligands, previously thought to be a ubiquitous characteristic of tubulin from higher eukaryotes. In this study, we sought to gain a better understanding of the structure-activity relationship of the colchicine site of this divergent isotype, using chicken erythrocyte tubulin (CeTb) as the model. We developed a fluorescence-based assay to detect binding of drugs to the colchicine site and used it to study the interaction of 53 colchicine-site ligands with CeTb. Among the ligands known to bind at this site, most colchicine derivatives had lower affinity for CeTb compared to brain tubulin. Remarkably, many of the benzimidazole class of ligands shows increased affinity for CeTb compared to brain tubulin. Because the colchicine site of human βVI tubulin is very similar to that of chicken βVI tubulin, these results may have relevance to the effect of anti-cancer agents on hematologic tissues in humans.

## 1 Introduction

Microtubules (MT) are subcellular structures whose arrays provide cells with structural rigidity, polarity, and mechanisms of intracellular transport. As such, they are central players in cell division, shape maintenance and changes, differentiation, and motility. Because of these multiple roles, MT have been targets of a large variety of therapeutic agents, binding to a number of known binding sites on the MT subunit protein, tubulin (Steinmetz and Prota, 2018).

Tubulin is a heterodimer composed of a non-covalent association of one alpha- and one beta-monomer. Multiple forms of each monomer exist in many species (such as humans), coded by multiple genes that produce very similar but non-identical proteins (isotypes) that are expressed in different levels in different tissues, and at different stages in development. Most MT-targeting ligands bind to sites on beta-tubulin, which have been considered to be largely the same in different isotypes. Therefore, measurements of protein drug binding (of colchicine, for example) has long been taken to be equivalent to measurements of active tubulin. However, some differences in the drug-binding properties of the β isotypes have been noted (Banerjee and Luduena, 1992).

The most divergent β tubulin isotype is known as β1, class VI (human protein is Q9H4B7), coded by the gene TUBB1, and has been studied less than other isotypes. We will refer to this as βVI tubulin, and is found in erythrocytes (Leandro-García et al., 2012), platelets (Feierbach et al., 1999), megakaryocytes (Lecine et al., 2000), as well as other sites such as brain and nasal epithelium (Palasca et al., 2018; Hausrat et al., 2021). Mice deficient in βVI have reduced levels of platelets, and the platelets they do have lack the characteristic discoid shape (Schwer et al., 2001; Italiano et al., 2003). Humans with mutations or deficits in TUBB1 reveal clotting disturbances and other disorders (Stoupa et al., 2018).

Nonmammalian red blood cells (RBCs), as well as immature mammalian RBCs such as human erythroblasts (Dmitrieff et al., 2017) contain a peripheral ring of MT that acts to maintain the ellipsoidal shape of the cells. Chicken (*Gallus gallus*) RBCs represent a readily available source of these MT, assembled from tubulin heterodimers that contain almost exclusively chicken βVI tubulin (P09207), coded by gene TUBB1 (NM_205445.2). We will refer to this (Chicken erythrocyte Tubulin) as CeTb. Previous studies with this tubulin have reported some differences from brain tubulin in binding of ligands, for example to the colchicine site (Sharma et al., 2010).

**FIGURE 1:**
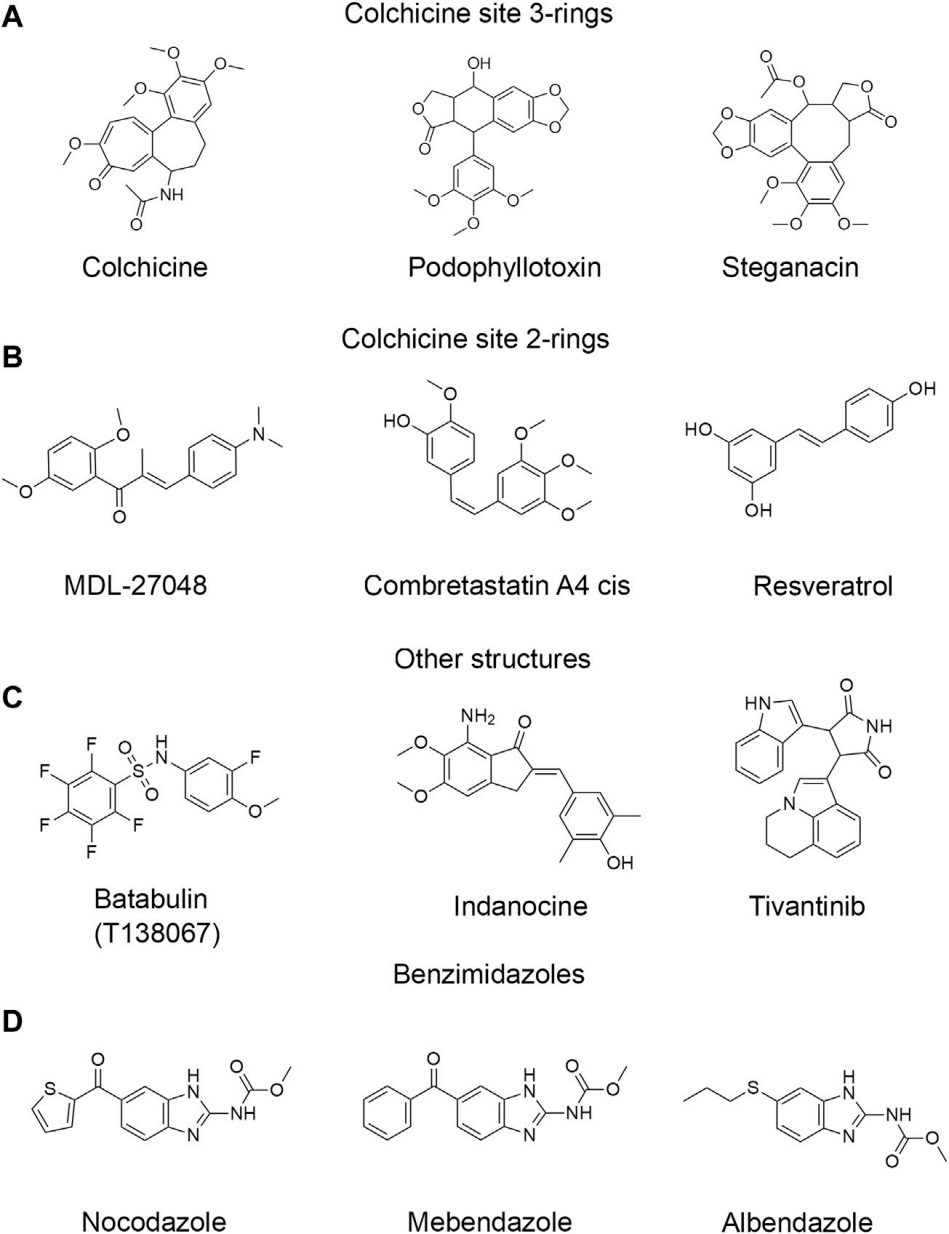
Structures of representative colchicine site drugs used in this work. **(A)** colchicine site 3-ring drugs: colchicine, podophyllotoxin and steganacin. **(B)** colchicine site 2-ring drugs: MDL, combretastatin A4 cis, resveratrol. **(C)** drugs with other non-colchicine structures: tubulazole C, indanocine, tivantinib. **(D)** benzimidazole drugs: nocodazole, carbendazim, oxibendazole. Full list of drugs structures is found in Supplementary Figures S1–S4.

In this work, we compared binding affinities of mammalian brain tubulins (BTb) and CeTb for 53 different ligands reported to bind at the colchicine site of tubulin, using a fluorescence-based competition assay. We use the term “BTb” for mammalian brain tubulins, typically from bovine or rat brain. When we specifically compare different brain tubulins we will specify the organism: rat brain tubulin—RBTb, or bovine brain tubulin—BBTb, or chicken brain tubulin—CBTb. The compounds selected for this survey (Figure 1 and Supplementary Figures S1–S4) include close colchicine analogs, as well as compounds whose structures bear no obvious relation to that of colchicine, but have been reported to bind to the colchicine site. Notable in this group are the benzimidazoles, whose extensive history as antihelmintic drugs in veterinary and human contexts have led to recent studies of repurposing for treatment of human cancers (Son et al., 2020).

## 2 Materials and Methods

### 2.1 Protein Purification

Rat brain tubulin was purified from microtubule protein previously extracted from frozen rat brains, as described previously (Sackett et al., 1991; Montecinos-Franjola et al., 2019). Tubulin from chicken brain and from chicken red blood cells was purified from frozen whole brains and from washed red blood cells (#33131-1, Pel-Freez Biologicals, Rogers, AZ) (https://www.pelfreez-bio.com/products/animal-serum-plasma-complement-and-ancillary-products/whole-blood-and-red-blood-cells/), respectively, as described previously (Sackett, 1995; Montecinos-Franjola et al., 2019). Bovine brain tubulin protein was from Cytoskeleton, Inc., Boulder, CO (#HTS02-A) or from Sigma-Aldrich, St. Louis, MO (#T4925). Purified proteins were stored in PM buffer (0.1M Pipes-KOH, pH 7.0, 1 mM MgCl_2_) at −80°C. The estimation of protein concentration was made using the Bradford assay (Bio-Rad) with BSA as the calibration standard (#23209, ThermoFisher Scientific, Waltham, MA).

### 2.2 Chemicals

MDL-(E)-1-(2,5-dimethoxyphenyl)-3-[4-(dimethylamino)phenyl]-2-methylprop-2-en-1-one (MDL) was not commercially available and therefore was synthesized according to the literature (Ducki et al., 1998) with the following minor modifications. In the second step, the oxidation of 1-(2,5-dimethoxyphenyl)-1-propanol using pyridinium dichromate, one equivalent of acetic anhydride was used per equivalent of starting material. The product from this reaction was stirred in diethyl ether with 2 g of Florisil per mmol of starting material and then filtered to remove excess pyridinium dichromate. The structure of the product was confirmed by 1H NMR. An extinction coefficient was measured in DMSO (ε_385 nm_ = 2.69 × 10^4^ M^−1^cm^−1^).

Drugs and test compounds were obtained from multiple sources listed in Supplementary Table S1. Concentrated stock solutions were prepared in water, in PM buffer or in DMSO as indicated in the same table, and stored at −20°C. The concentration was determined spectrophotometrically for those compounds with known extinction coefficients.

### 2.3 Polymerization Kinetics

Tubulin was polymerized at a concentration of 2 µM in PM buffer (0.1 M Pipes pH 7, 1 mM MgCl_2_) + 0.1 mM GTP in a volume of 60 µL. The reaction mixture was transferred to a 10-mm pathlength microcuvette (Hellma United States Inc., Plainview, NY) and placed in the cuvette holder of a SpectraMax M2 Multimode Microplate Reader (Molecular Devices, San Jose, CA) equipped with temperature control set at 37°C for these experiments. Drugs at 20 µM were added directly to the polymerization mixture and incubated for 15 min previous to starting the recordings. Tubulin polymerization kinetics was followed by recording the absorbance at 350 nm. After recording the baseline, tubulin polymerization was induced by the addition of 2 µM paclitaxel (taxol). The final concentration of DMSO in the mixtures was 4%–5% v/v.

### 2.4 Fluorescence Measurements

The fluorescence emission of MDL was recorded at 550 nm in the SpectraMax M2 Multimode Microplate Reader with excitation at 400 nm. Tubulin samples at 1 µM were prepared in PM buffer in 60 µL volume, incubated for 30 min, and then transferred to a 10-mm pathlength microcuvette which later was placed in the cuvette holder of the SpectraMax M2 set at 25°C. The kinetics of MDL binding and its displacement by competitor drugs was detected by recording the fluorescence emission at 550 nm every 5 s. MDL was used at 10 µM and podophyllotoxin at 100 µM. For fluorescence spectra recordings, the drugs were added at 50 µM final prior to addition of MDL and the DMSO was adjusted to 5% v/v.

### 2.5 Determination of Dissociation Constants and Competition Experiments

The equilibrium dissociation constants for the binding of MDL to tubulin were determined from the concentration-dependent changes in the fluorescence emission at 550 nm upon serial dilution of the MDL. The starting mix contained 1 µM tubulin and 10 µM MDL all in PM buffer (starting mix). The dilution mix contained 1 µM tubulin in PM buffer and was prepared in a larger volume (dilution mix). The concentration of DMSO was adjusted to 5% v/v in both cases. The dilution series was prepared by mixing the “starting mix” and the “dilution mix” at a 1:3 ratio resulting in a decrease of the MDL concentration to 1/3 of the original, or 3.3 µM, while keeping the tubulin concentration constant at 1 µM. This new sample was then mixed at 1:3 ratio with the “dilution mix” for obtaining the next sample in the dilution series at 1.1 µM MDL. Subsequent samples in the dilution series were prepared in the same fashion until reaching a low concentration <1 nM MDL. Next, 60 µL of the dilution series were transferred to a 96-well black half-area flat bottom fluorescence microplates (#3642 Corning, NY) including the blanks. The loaded plates were incubated for at least 30 min and then placed in the SpectraMax M2 Multimode Microplate Reader for recording the fluorescence signals with temperature control set at 25°C.

For competition experiments with colchicine-site drugs, the samples were prepared in a similar fashion with some changes. For full dilution series experiments (e.g., podophyllotoxin and benzimidazoles), the starting mix contained 1 µM tubulin and 5 µM MDL all in PM buffer (starting mix). The dilution mix contained 1 µM tubulin, 5 µM MDL and 100 µM drugs, all in PM buffer, and was prepared in a larger volume (dilution mix). The concentration of DMSO was adjusted to 8% v/v in both cases to promote the solubility of the ligands. The dilution series was prepared by mixing equal amounts (1:1) of each mix resulting in a 50% decrease of the initial drug concentration at each step of the dilutions series, while keeping constant the concentrations of tubulin at 1 µM and of MDL at 5 µM. For the single concentration drug screening experiments, the samples contained 1 µM tubulin (unless otherwise specified), 5 µM MDL and the drugs were added at a final concentration of 50 µM directly to the mixtures. Next, 60 µL of the samples were transferred to 96-well microplates. For competition experiments, the loaded plates were incubated for at least 1 h due to the slower kinetics observed for MDL release from the colchicine binding site after addition of the competing drugs. The MDL fluorescence was recorded in the SpectraMax M2 Mulitmode Microplate Reader with temperature control set at 25°C.

### 2.6 Data Analysis

The dissociation constant for the MDL-tubulin interaction was determined by fluorescence intensity measurements as previously described for MDL and other colchicine binding-site drugs (Peyrot et al., 1992; Sharma et al., 2010). The raw fluorescence signal was blank-subtracted and plotted as a function of the drug concentration. The data was analyzed by non-linear regression using the following one-component binding model that relates the fluorescence intensity observed (I_obs_) with the apparent equilibrium dissociation constant K_d,MDL_ as:

$${\text{K}}_{\text{d},\text{MDL}}=\;\frac{\left[\text{Tub}\right]\cdot \left[\text{MDL}\right]}{\left[\text{Tub}-\text{MDL}\right]}$$ (1)

$${\text{I}}_{\text{obs}}=\;\frac{{\text{I}}_{\text{free}}+\left(\frac{\left[\text{MDL}\right]}{{\text{K}}_{\text{d},\text{MDL}}}\cdot {\text{I}}_{\text{bound}}\right)}{1+\frac{\left[\text{MDL}\right]}{{\text{K}}_{\text{d},\text{MDL}}}}$$ (2)

Where (Tub) and (MDL) are the concentrations of free tubulin and MDL at equilibrium, I_free_ and I_bound_ are fluorescence signals of the free MDL in solution and of MDL bound to tubulin under saturating concentrations, respectively. Weights were assigned to each data point based on the reciprocal of the standard deviation of each data point (averages of three to four measurements). For comparing plots of the various tubulins, or the different drugs, the data was converted to fraction bound with the following relationship:

$${\text{f}}_{\text{b}}=\;\frac{{\text{I}}_{\text{obs}}-{\text{I}}_{\text{free}}}{{\text{I}}_{\text{bound}}-{\text{I}}_{\text{free}}}$$ (3)

In the competition experiments, for instance with podophyllotoxin, the value of IC_50_ was determined graphically from plots of f_b_ vs. (podophyllotoxin) and the value of the apparent dissociation constant, K_d,podo_ was calculated with the following equation (Yung-Chi and Prusoff, 1973; Craig, 1993):

$${\text{K}}_{\text{d},\text{podo}}=\;\frac{{\text{IC}}_{50}}{1+\frac{\left[\text{MDL}\right]}{{\text{K}}_{\text{d},\text{MDL}}}}$$ (4)

For single point competition experiments, the F/F_max_ ratios were calculated from the fluorescence intensity values recorded in the presence of the competitor drug, F, and the fluorescence intensity of MDL in the absence of competitor, F_max_. The percentage inhibition was calculated from the F/Fmax ratios:

$${\text{\%}}_{\text{inhibition}}=\left(1-\frac{F}{F_{max}}\right)\times 100$$ (5)

All of the competition titrations yielded curves consistent with simple single-site binding, and therefore we make the assumption that all of the tested compounds do as well. With this assumption, single point data (50 µM test compound) was fit with a single-site binding isotherm by non-linear regression. Then the mid-point of that curve (IC_50%_) was used to calculate the K_d(app)_ using Eq. 4, as above. The curve-fitting procedure did not converge for % inhibition below 1%, therefore the apparent dissociation constant could not be determined in those cases (nd). For further details and examples, see Supplementary Material.

## 3 Results

### 3.1 Interaction of a Fluorescent Probe With CeTb

**FIGURE 2:**
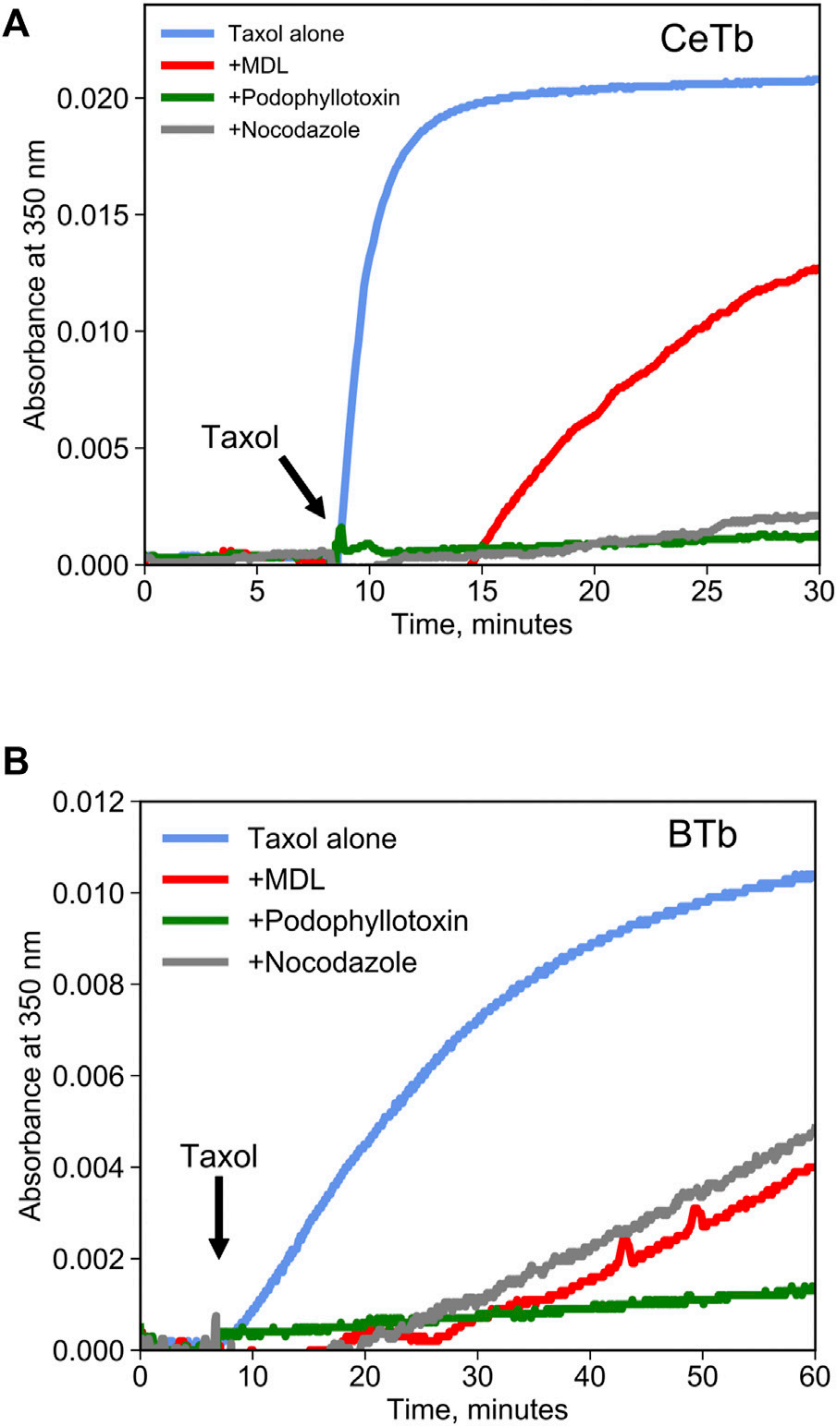
Inhibition of tubulin polymerization by colchicine-site drugs. Tubulin from brain **(A)** or from erythrocytes **(B)** was incubated at 2 µM in polymerization buffer at 37°C, and the absorbance at 350 nm was registered for 10 min prior to starting the polymerization by the addition of taxol 2 µM (arrow). Drugs were included in polymerization buffer and used at a concentration of 20 µM.

Competition binding assays are often used as a method to quantify binding to a target protein. Such assays can be performed using a tritiated ligand that is known to bind to the target, but spectroscopic methods are more convenient. Colchicine is often used for competition binding studies on tubulin, but it does not bind well to CeTb so it cannot be used for the purposes of this study. Another molecule that is a candidate for a competition binding assay is known in the literature as MDL-27048 (Figure 1). The fluorescence of MDL-27048 (hereafter referred to as MDL) is very weak when free in solution and greatly enhanced upon binding to the colchicine site of mammalian brain tubulin (BTb) (Peyrot et al., 1992). However, it was unknown whether MDL would interact with CeTb. Colchicine-site drugs inhibit the polymerization of purified tubulin into microtubules. In assays using tubulin from mammalian brain, MDL has been found to inhibit polymerization (Peyrot et al., 1989). Therefore, we polymerized CeTb in the presence of MDL to look for inhibition. We found that MDL does inhibit the polymerization of CeTb (Figure 2; quantitation is in Table 1), indicating that it does interact with CeTb.

**TABLE 1:** Inhibition of tubulin taxol-induced polymerization by colchicine-site drugs.

Drug	Brain tubulin (BTb) % inhibition at 60 min	Erythrocyte tubulin (CeTb) % inhibition at 30 min
Taxol (control)	0	0
MDL	64	38
Podophyllotoxin	90	95
Nocodazole	55	90
Mebendazole	92	25

Polymerization in presence of taxol alone was considered the 100%.

Tubulin was used at 2 μM, Taxol at 2 µM and drugs were used at 20 µM in polymerization buffer at 30°C.

**FIGURE 3:**
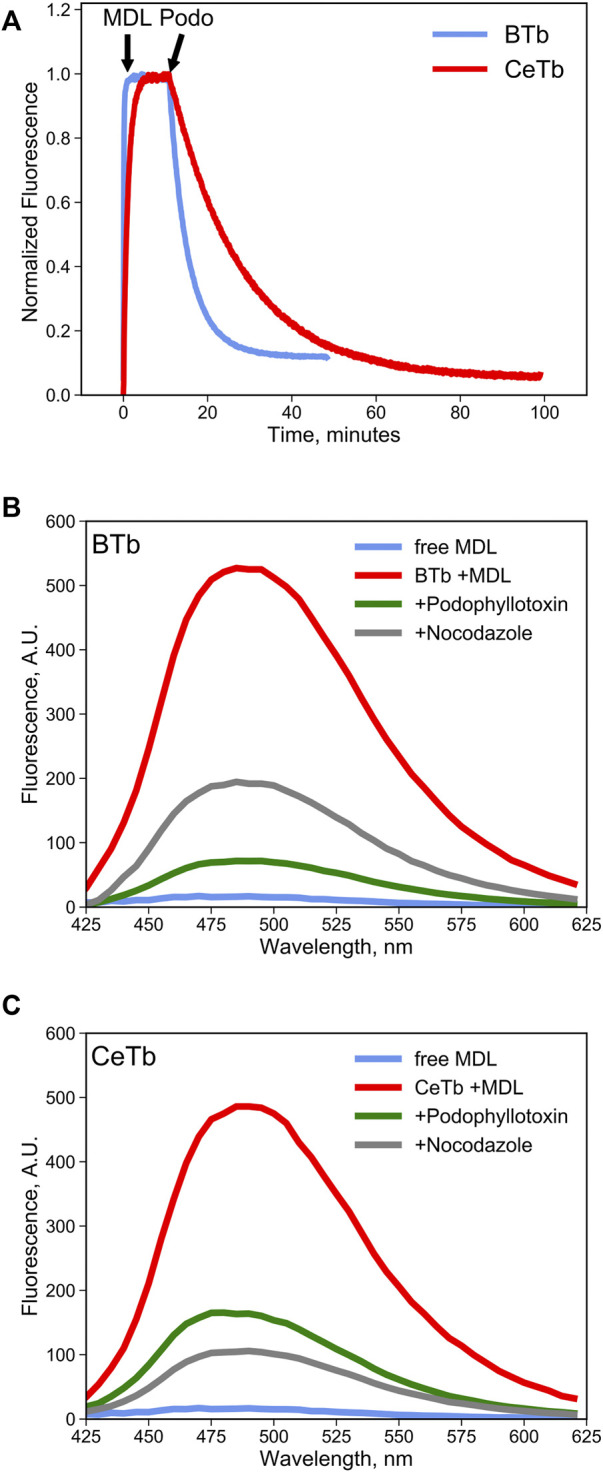
MDL binding to tubulin and competition by colchicine-site drugs. **(A)** the fluorescence enhancement of MDL upon binding to tubulin was used to follow the kinetics of the interaction. Fluorescence was recorded at 550 nm every 5 s with excitation at 400 nm. Tubulin 1 µM was incubated in PM buffer at room temperature for at least 30 min prior to addition of the drugs at the indicated times (arrows). MDL was used at 10 µM and podophyllotoxin at 100 µM. **(B,C)** fluorescence emission spectra of free MDL in solution or bound to brain **(B)** tubulin or to erythrocyte **(C)** tubulin in PM buffer at 25°C. Colchicine-site drugs were added at 50 µM final concentration in PM buffer prior to addition of 10 µM MDL. The decrease in MDL fluorescence is evidence the competitive binding by the selected drugs that displaces the fluorescent molecule from the colchicine site in tubulin.

It is reasonable to suppose that MDL binds to the colchicine site on CeTb, as it does to BTb, resulting in inhibition of polymerization of both tubulins. To be useful in a competition binding assay, it must also exhibit a spectroscopic change upon binding. We therefore looked for a change in the fluorescence of MDL in the presence of tubulin, including CeTb. The emission spectrum showed a large enhancement of fluorescence upon binding to CeTb, as well as to BTb (Figure 3). Fluorescence intensity increases rapidly upon addition of MDL to BTb or CeTb, and is then displaced by addition of 10-fold excess of the colchicine site ligand podophyllotoxin (structure in Figure 1). Displacement kinetics differ between BTb and CeTb, indicating a slower off-rate for MDL from CeTb compared to BTb. Addition of the benzimidazole nocodazole also displaces MDL, confirming the usefulness of this assay for binding of diverse compounds.

**FIGURE 4:**
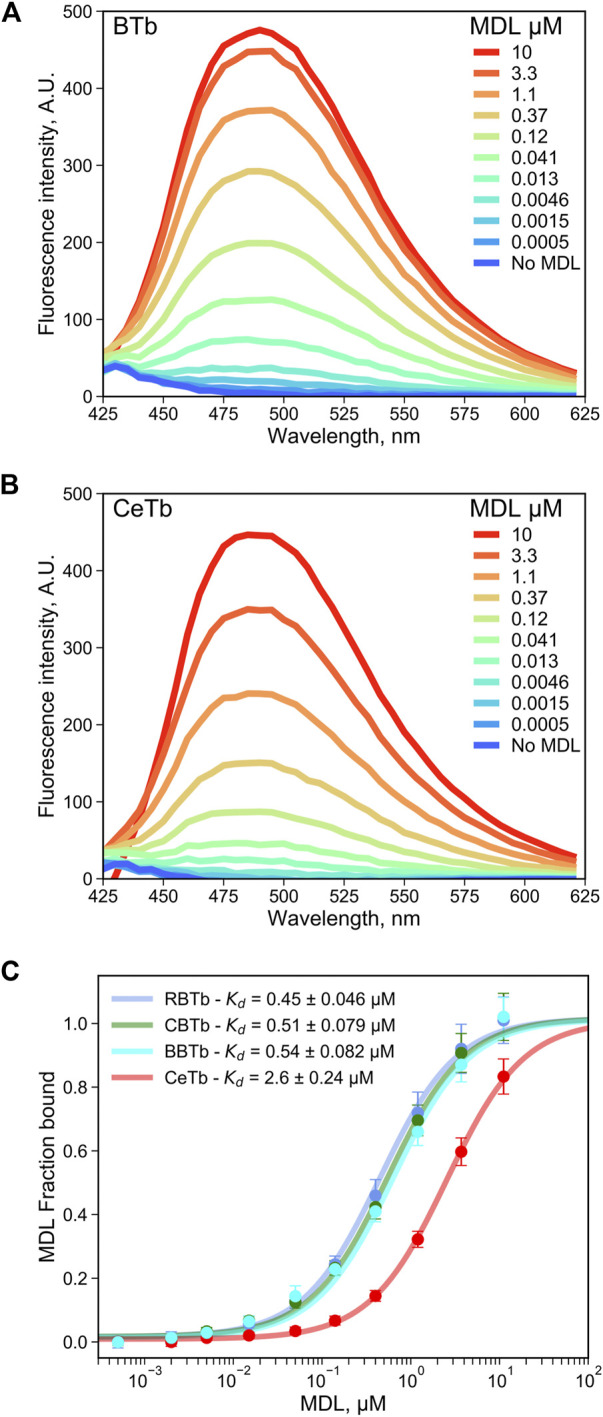
Determination of the dissociation constant for tubulin—MDL interaction. The fluorescence enhancement of MDL upon binding to tubulin from brain **(A)** or from erythrocytes **(B)**, both at 1 μM, was monitored by recording the fluorescence emission spectra with excitation at 400 nm. **(C)** the MDL concentration-dependent change in the fluorescence intensity at 550 nm was used to determine the dissociation constant by non-linear regression using Eq. 1. The binding isotherms of tubulin extracted from chicken erythrocytes (CeTb) is compared with tubulin extracted from rat brain (RBTb), from chicken brain (CBTb and from cow brain (bovine, BBTb). The data points are the averages of three to four samples and the error bars are standard deviations. The best-fit K_d_ are shown with the standard errors of the fits.

This emission enhancement allowed us to determine the binding affinity of MDL for CeTb, compared to brain tubulin from chicken brain (CBTb), rat brain (RBTb), and bovine brain (BBTb) (Figures 4A,B). Tubulins were titrated with MDL, and the concentration dependence of the emission signal was used to determine the affinity. The resulting binding isotherms are shown in Figure 4C and the fitted K_d_ values for binding are presented in Figure 4C and Table 2. It is notable that all the brain tubulins, including CBTb, yield very similar Kd for MDL binding to the protein, while MDL binding to CeTb shows affinity ∼5–fold weaker than to brain tubulins.

**TABLE 2:** Apparent dissociation constants for the Tubulin—MDL interaction measured by dilution experiments.

Tubulin source	K_d,MDL_, µM
Chicken brain (CBTb)	0.51 ± 0.079
Rat brain (RBTb)	0.45 ± 0.046
Bovine brain (BBTb)	0.54 ± 0.082
Chicken Erythrocytes (CeTb)	2.6 ± 0.24

Uncertainties are the standard errors of the fits from Figure 4.

**FIGURE 5:**
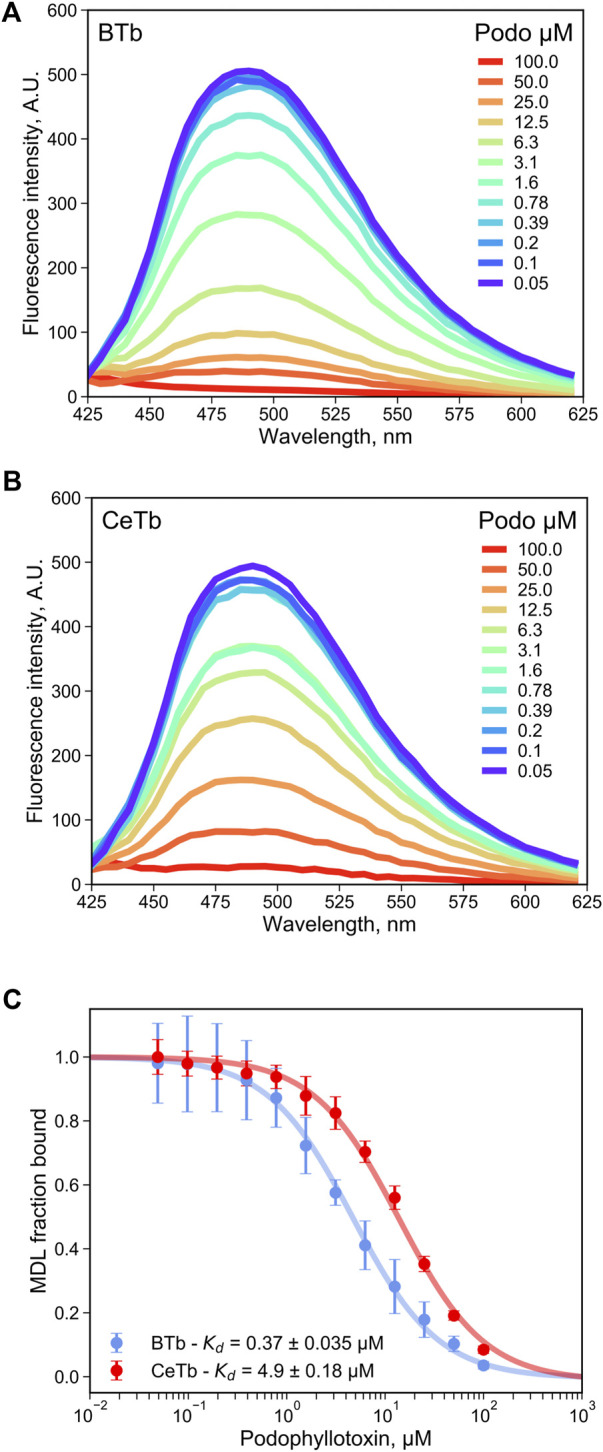
Determination of the dissociation constant for tubulin—podophyllotoxin interaction by competition experiments. The fluorescence of tubulin-bound MDL gradually decreases upon displacement by the competitor podophyllotoxin as monitored by the fluorescence emission spectra with excitation at 400 nm, for tubulin from brain **(A)** or from erythrocytes **(B)**. **(C)** the podophyllotoxin concentration-dependent change in the fluorescence intensity at 550 nm was used to determine the apparent dissociation constant by non-linear regression using Eq. 4. 1 µM tubulin with 5 µM MDL was incubated in PM buffer at room temperature at the indicated podophyllotoxin concentrations. The data points are the average of three to four measurements and the error bars are standard deviations. The uncertainties of K_d_ values are the standard error of the fits.

As a test of utility of MDL in a competition assay for ligand binding to the colchicine site, the binding of the reversible colchicine site ligand podophyllotoxin was chosen. It was shown in Figure 2 that excess podophyllotoxin could displace MDL from both BTb and CeTb, so we used titration of tubulin- MDL with increasing concentrations of podophyllotoxin to measure displacement by loss of fluorescence intensity. The resulting data for BTb and for CeTb are shown in Figures 5A,B, respectively. Displacement as a function of podophyllotoxin concentration is shown in Figure 5C, and the derived K_d_ for binding of podophyllotoxin to BTb and to CeTb (calculated as described in Materials and Methods) are given in the inset to Figure 5C and in Table 3. The K_d_ for podophyllotoxin binding to BTb was found to be ∼0.5 μM, essentially the same as the literature value of 0.55 µM found in rat brain tubulin using tritiated podophyllotoxin (Cortese et al., 1977). The K_d_ for CeTb, ∼6 µM indicates about a 12-fold weaker binding of podophyllotoxin for CeTb compared to BTb.

**TABLE 3:** Dissociation constants of tubulin—benzimidazoles-, podophyllotoxin-, and combretastatin A4 - interaction measured by competition titration with MDL.

Drug	Brain tubulin (BTb)		Erythrocyte tubulin (CeTb)	
	IC_50_, µM	K_d_, µM	IC_50_, µM	K_d_, µM
Nocodazole	11 ± 1.2	1.0 ± 0.11	1.5 ± 0.11	0.51 ± 0.036
Mebendazole	14 ± 2.5	1.3 ± 0.23	6.8 ± 0.73	2.3 ± 0.25
Carbendazim	nb	nb	37 ± 5.4	13 ± 1.8
Oxibendazole	nb	nb	36 ± 5.6	12 ± 1.9
Albendazole	nb	nb	104 ± 12	35 ± 4.0
Podophyllotoxin	4.0 ± 1.0	0.37 ± 0.035	15 ± 0.53	4.9 ± 0.18
Combretastatin A4	2.6 ± 0.22	0.13 ± 0.011	20 ± 1.2	5.7 ± 0.32

K_d_ was calculated with Equation 4.

Uncertainties are the standard errors of the fits. nb, no binding detected.

A similar analysis was performed using the compound cis-Combretastatin A4 (structure in Figure 1), This two-ring analog of colchicine binds with considerable affinity as well as specificity (the trans-form is notably less potent) and has been the subject of much preclinical development (Cushman et al., 1991) [reviewed in (Sackett, 1993)]. Titration of CeTb- and BTb-bound MDL with combretastatin A4 yielded a K_d_ of 5.7 ± 0.32 µM for CeTb, while the K_d_ for BBTb was found to be 0.13 ± 0.011 µM (Supplementary Figure S5) The K_d_ for BBTb is similar to the literature value of 0.12 µM (Lin et al., 1989). Results are included in Table 3, and indicate a ∼44-fold weaker binding to CeTb compared to BTb.

**FIGURE 6:**
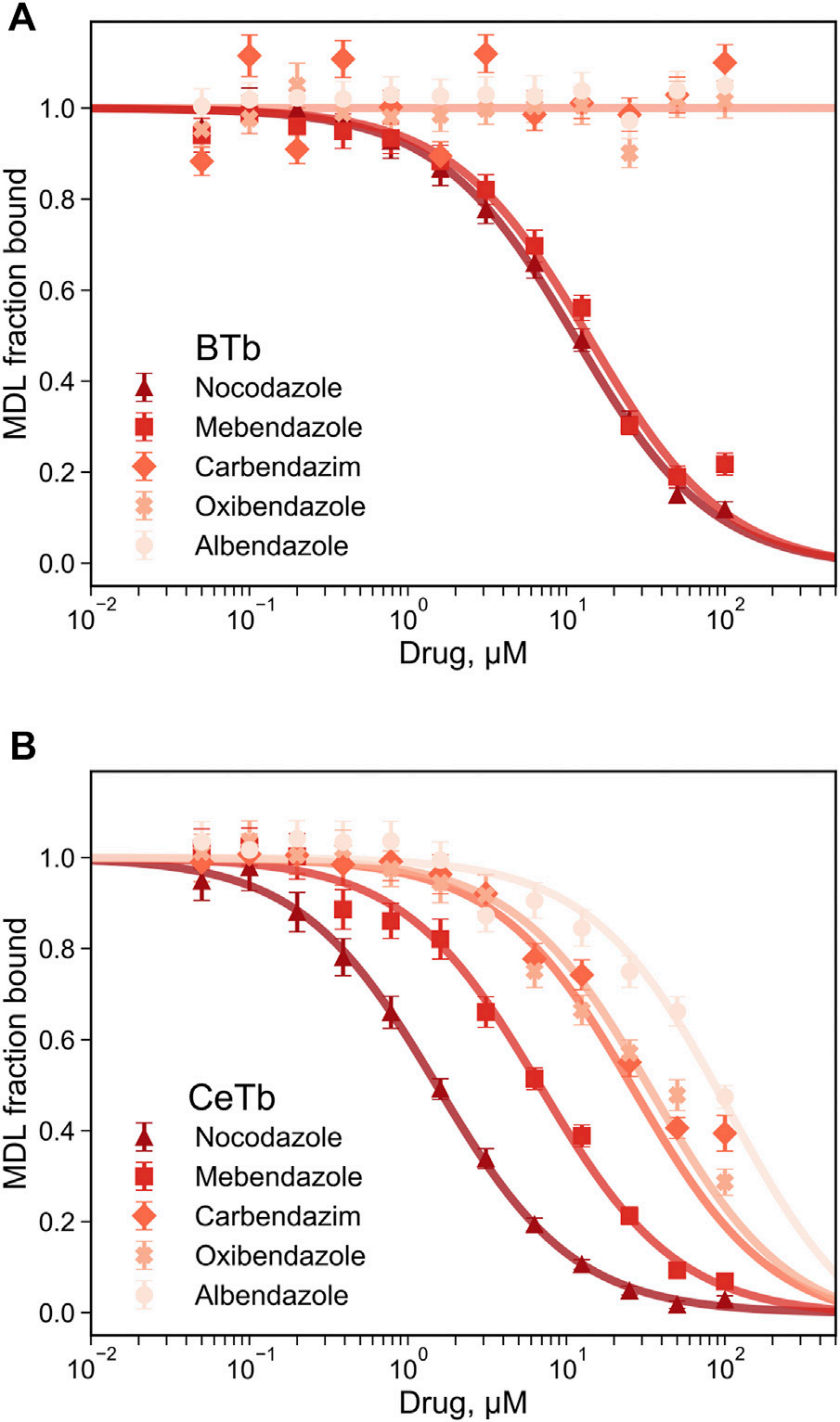
Determination of the dissociation constant for tubulin—benzimidazoles interaction by competition experiments. The fluorescence of tubulin-bound MDL gradually decreases upon displacement by the benzimidazoles competitors. Fluorescence was monitored at 550 nm with excitation at 400 nm, for tubulin from brain **(A)** or from erythrocytes **(B)**. The reaction mixtures contained 1 µM tubulin with 5 µM MDL incubated in PM buffer at room temperature at the indicated drug concentrations. The data points are the average of three to four measurements and the error bars are standard deviations. The best-fit K_d_ with the standard errors of the fits are shown in Table 3.

Since fluorescence titration and competition assays with MDL and two standard compounds yielded binding values consistent with literature values with BTb, we accept the values obtained for CeTb as well and turned our attention to other compounds using this assay. The first group of compounds we examined were the benzimidazoles. These compounds have a history as antihelmintics that target parasite tubulin over mammalian host tubulin, but are causing renewed interest as potential repurposed agents in human oncology (Son et al., 2020). Five compounds were chosen for a full competition titration against CeTb- and BTb-bound MDL. The titration curves and best fits to the data are shown in Figure 6, and the obtained K_d_ values are presented in Table 3. The data show that nocodazole yields a Kd of about 1 µM to BTb, similar to the published value of 2.5 µM (Head et al., 1985). Mebendazole binding to BTb is similar, and both compounds have similar Kd (within a factor of two) with either tubulin. Other compounds (albendazole, oxibendazole, carbendazim) show moderate binding affinity with CeTb, but no detectable binding to BTb.

The dissociation constants obtained for selected compounds are combined in Table 4 for both CeTb and BTb. The last column shows the ratio of the drug’s K_d_ for binding to CeTb compared to that for BTb. A ratio >1 (i.e., K_d_ for CeTb > K_d_ for BTb) indicates weaker binding to CeTb than to BTb. It is clear that the relative affinity for the two tubulins shows considerable variation, with the greatest differential in the first three “standard” colchicine site compounds found with Combretastatin A4, the strongest binding ligand for BTb, showing 44-fold lower affinity for CeTb. In the benzimidazoles, however, the range of K_d_ ratios is even broader, since two of the compounds do not show binding to BTb at all under these assay conditions.

**TABLE 4:** Dissociation constants of selected drugs with CeTb and BTb.

Drug	Erythrocyte tubulin (CeTb) K_d_, µM	Brain tubulin (BTb) K_d_, µM	Ratio^a^
MDL	2.6	0.51	5
Podophyllotoxin	4.9	0.37	13
Combretastatin A4	5.7	0.13	44
Nocodazole	0.6	1.1	0.5
Mebendazole	2.6	1.4	1.9
Albendazole	35	nd^b^	nd
Oxibendazole	12	nd	nd

a The last column (“Ratio”) shows the K_d_ of the drug and CeTb divided by the K_d_ of the drug and BBTb.

b nd, not determined.

### 3.2 Screen of Known Colchicine-Site Ligands

Next, we used this method to screen a single concentration of a series of molecules. Many colchicine-site ligands are known, so these were an excellent starting point to look for drugs that have activity in CeTb. A series of known colchicine site drugs was obtained and screened for binding to CeTb. Some compounds are structurally close to colchicine, others not so similar, including benzimidazoles. The molecules studied above, podophyllotoxin and combretastatin, were included in the screen. The results for these two drugs were consistent with the full binding curves obtained above, so we felt that valuable information about the interaction of the various drugs with CeTb (and BTb) could be obtained from a single concentration reading. For this purpose, we used a relatively high concentration of test compounds in order to probe weaker as well as stronger binding.

The results of the single-point screen are shown in Tables 5–8. The test compounds are divided into the following groupings: three-ring colchicine analogs, two-ring colchicine analogs, a group of diverse colchicine-site compounds which are structurally unrelated to the colchicine groups, and the benzimidazoles. Information about the compounds is given in Supplementary Table S1 and structures in Supplementary Figures S1–S4.

**TABLE 5:** MDL competitive binding inhibition by colchicine site 3-ring drugs.

#	Drug	BTb %	BTb K_d(app)_, µM	CeTb %	CeTb K_d(app)_, µM
1	Colchicine	58	3.2	24	54
2	Colcemid	60	3.0	28	44
3	Deacetylcolchicine (DAC)	26	13	11	138
4	Desacetamidocolchicine (DAAC)	76	1.4	38	28
5	Isocolchicine	1	502	3	565
6	Cornigerine	67	2.2	36	30
7	Steganacin	50	4.5	50	17
8	Allocolchicine	52	4.2	28	44
9	Allo methyl ketone	93	0.34	86	2.7
10	Allo ethyl ketone	92	0.39	89	2.1
11	Thiocolchicine	43	6	31	38
12	1,2-Didemethylcolchicine	11	37	6	269
13	Podophyllotoxin	90	0.73	81	5.9
14	Azatoxin	<1	nd	4	416
15	Colchiceine	11	37	10	153
16	Trimethylcolchicinic acid	22	16	24	54

nd, not determined

A comment about this assay is in order. The assay measures the inhibition of MDL binding to CeTb and BTb by a single concentration of test compound. Since the two tubulins have 5-fold difference in K_d_ for MDL, a reduction in MDL binding by an identical fraction will imply different binding strengths in the two tubulins. Since MDL binding to CeTb is weaker than to BTb, it will be more easily competed away. Thus, with fixed tubulin and MDL concentrations of 1 and 5 μM, respectively, a competitor/compound that caused a 50% reduction in MDL binding to BTb at 50 μM, would imply a Ki of ∼4.5 µM, while a 50% reduction in MDL binding to CeTb by 50 µM competitor would imply a K_i_ of ∼17 µM. Since all of the drugs analyzed here by titration (Figures 4–6) yielded curves that indicate simple single-site binding, we analyzed the single concentration data using a single-site binding isotherm by non-linear regression. As with the titrations, this curve was used to obtain the IC_50%_ which served to calculate an apparent K_d_ (K_d(app)_) using Eq. 4 (for more details see Section 2 and Supplementary Figure S6).

The drugs in the colchicine series (Table 5) displayed weak binding to CeTb, and in general notably lower binding to CeTb than to BTb. Isocolchicine does not bind to either tubulin, demonstrating that the stereospecific requirements for binding to CeTb mimic those of BTb. Interestingly, steganacin inhibits MDL binding to CeTb to the same extent (%) as to BTb (though note that this still indicates a lower affinity/higher K_d(app)_ to CeTb than to BTb). The compounds that show the strongest binding to CeTb are the same ones that bind strongest to BTb: allocolchicine methyl ketone and allocolchicine ethyl ketone. Both compounds bind to both tubulins more strongly than does the parent compound, allocolchicine, or colchicine itself. While both compounds significantly inhibited MDL binding to both tubulins, the binding to CeTb is weaker than to BTb. By comparison to podophyllotoxin, the K_d(app_) for binding of both compounds to both tubulins can be estimated to be lower by a factor of about two than that obtained by titration with podophyllotoxin. This shows that small modifications to the structure of a colchicine-site ligand can improve binding to both tubulins. Of interest here is a rational drug design study by Paré et al. (2020) who showed that some small changes to colchicine yielded a lead compound with increased affinity for βVI tubulin and increased bioactivity towards neutrophils.

Data from the two-ring colchicine analogs (Table 6) demonstrate the greater relative importance of the cis configuration of the two rings in CeTb compared to BTb. This can be seen by comparing combretastatin-A2 and -A4 (cis), both of which have an unsaturated bridge holding the two rings in cis, with combretastatin-A4 (trans) and dihydrocombretastatin-A4, in which the rings are locked in trans, or are freely rotating, respectively. The potency of the two cis compounds as competitors approaches that of podophyllotoxin, and is only slightly less than the two allocolchicine analogs in Table 5. The lack of activity of trimethoxy resveratrol (trans) is consistent with this observation.

**TABLE 6:** MDL competitive binding inhibition by colchicine site 2-ring drugs.

#	Drug	BTb %	BTb K_d(app)_, µM	CeTb %	CeTb K_d(app)_, µM
17	AC (MTC)	16	24	3	565
18	Combrestastatin (CS) A2	91	0.44	81	3.9
19	CS-A4 cis	92	0.39	64	9.5
20	CS-A4 trans	44	5.8	7	228
21	Dyhydro CS A4	46	5.3	10	153
22	Trimethoxy resveratrol (trans)	8	53	28	43
23	Resveratrol	2	235	4	416
24	MDL	nd	nd	nd	nd

nd, not determined

The compounds of diverse structure in Table 7 yielded only two compounds with significant binding to CeTb. Indanocine shows similar inhibition of MDL binding to BTb and to CeTb, comparable to steganacin in Table 5, consistent with a ca. 4.5-fold difference in K_d(app)_. D-64131 shows the highest inhibitory potency to both tubulins of all the compounds in this table, while still showing an approximately 10-fold ratio in K_d(app)_. Binding potency is similar to podophyllotoxin (for BTb) and slightly less so for CeTb. The largest differential between the tubulins appears to be with lexibulin, which is somewhat less potent than podophyllotoxin with BTb but nearly inactive with CeTb.

**TABLE 7:** MDL competitive binding inhibition by other non-colchicine structure drugs.

#	Drug	BTb %	BTb Kd (app), µM	CeTb %	CeTb Kd (app), µM
25	Tubulazole C	31	10	10	153
26	Tubulazole T	<1	nd	12	124
27	Indanocine	57	3.4	50	16
28	T113242	44	5.8	2	872
29	T138067 (Batabulin)	36	8.0	4	415
30	ABT-751	44	5.8	12	124
31	TN16	74	1.5	34	32
32	Tivantinib	48	4.9	24	53
33	Plinabulin	<1	nd	<1	nd
34	Lexibulin	80	1.1	6	268
35	Curvulin	3	152	2	872
36	Indibulin (D248510)	7	61	10	153
37	Curvularin	<1	nd	<1	nd
38	Dehydrocurvularin	2	234	1	1865
39	Tryprostatin	<1	nd	<1	nd
40	2-methoxyestradiol (2-ME)	12	33	1	1865
41	Berberine	4	111	2	872
42	D-64131	90	0.5	73	6.2
43	Ferulenol	10	41	2	872

nd, not determined.

Benzimidazole compounds (Table 8) yield several patterns of comparative inhibition. Nocodazole and mebendazole are fairly potent inhibitors of MDL binding to both tubulins, comparable to podophyllotoxin. Both compounds yielded Kd (app) values within a factor of two of the Kd values obtained from titrations, with both tubulins. Three compounds show moderate inhibition of MDL binding to both tubulins, but slightly higher % inhibition to MDL-CeTb than to MDL-BTb: fenbendazole, flubendazole, and benomyl [Kd (app) values are within a factor of two for the two tubulins]. A final group of five shows no or very low inhibition of MDL binding to BTb (<10%), but moderate to significant inhibition of MDL-CeTb: thiabendazole, carbendazim, albendazole, ricobendazole, and oxibendazole. We were somewhat surprised at the lack of inhibition of MDL binding by these compounds, especially albendazole, which has shown activity in mammalian cells that has prompted discussion of repurposing this compound for cancer therapy (Nath et al., 2020; Will Castro et al., 2021). We were unable to find a published study of direct binding of albendazole or these other benzimidazoles to BTb, and therefore we checked albendazole for direct inhibitory activity against polymerization of BTb. We observed little or no inhibition of polymerization of BTb in the albendazole concentration range that we have been studying (Supplementary Figure S7), consistent with the lack of inhibition of MDL binding that we observed in the titration and single-point assays.

**TABLE 8:** MDL competitive binding inhibition by benzimidazole drugs.

#	Drug	BTb %	BTb Kd (app), µM	CeTb %	CeTb Kd (app),µM
44	Nocodazole	85	0.8	96	0.68
45	Mebendazole	82	1.0	90	1.8
46	Thiabendazole	6	72	32	35
47	Carbendazim	<1	nd	58	12
48	Fenbendazole	12	33	39	26
49	Flubendazole	46	5.3	61	10
50	Albendazole	<1	nd	35	31
51	Ricobendazole	8	52	27	45
52	Oxibendazole	<1	nd	56	13
53	Benomyl	34	8.8	45	20

nd, not determined

## 4 Discussion

These findings expand our knowledge of the structure-activity relationship of the colchicine site on CeTb, and demonstrate the utility of the MDL competition assay for testing colchicine site ligands. This assay could readily be used for other compounds. It could also be used for high throughput screening of other types of tubulins for colchicine-site drugs. In the current application, this assay readily provided binding information via competition assay, due to the significant difference in fluorescence between the tubulin-bound and free forms of MDL.

For most compounds in this study, binding to CeTb is weaker than to BTb, to an extent that varies considerably. Perhaps this is due to these compounds having been selected for interest initially due to bioactivity against cells that express the beta tubulin isotypes in brain tubulin rather than those in erythrocyte tubulin. In any case most of the compounds from all groups that we studied did show reasonable binding to CeTB, and some bind more tightly to CeTb than to BTb. Notable in this regard are the benzimidazoles, about half of which showed significant binding to CeTb but no measurable binding to BTb under the conditions of our assay.

Previous studies showed thiabendazole to be a good inhibitor of nematode tubulin while it had virtually no effect on mammalian tubulin assembly, an observation which is consistent with our data demonstrating no MDL-BTb inhibition by thiabendazole (Dawson et al., 1984). Other studies (e.g., Lubega and Prichard, 1991) showed significant differences between benzimidazoles in binding to parasite tubulin, and also noted that some compounds showed significantly lower tubulin binding than expected from their known antihelmintic potency, possibly indicating the importance of other targets in bioactivity as well as tubulin (Shrivastava et al., 2017). Given the interest in repurposing several members of this family of compounds for human cancer therapy, a systematic direct study of benzimidazole binding to mammalian tubulin combined with a parallel measure of bioactivity such as inhibition of growth of human cell lines in culture would be a valuable addition to this field.

These findings may have relevance to human cancer. Chicken βVI and human βVI have a high degree of similarity in colchicine-binding region (Sharma et al., 2010). As we show in Supplementary Figure S8, of the 38 amino acid residues that fall within 6 Å of the bound colchicine, only one residue is substituted in chicken βVI compared to human βVI (I236V). Sequence alignments comparing βVI from chicken and human (and rat) are presented in Supplementary Figure S8. This also compares these sequences with the sequence of TUBB2B, the major β-tubulin in mammalian brain tubulin. Additionally, Supplementary Figure S8 shows the colchicine binding site of TUBB2B and indicates the residues that differ in TUBB2B and TUBB1.

βVI tubulin has long been recognized to be present in megakaryocytes and platelets (Lewis et al., 1987), as well as in other blood cells (Leandro-García et al., 2012). Thus, study of βVI tubulin may aid in the development of drugs for cancers of a variety of hematologic tissues. In particular, the activity of some of the benzimidazoles against this isotype may be informative in the effort to repurpose these widely used antiparasite drugs to use in human disease, and to enhance effectiveness of ligands at this site in treatment of inflammatory diseases.

## Data Availability

The original contributions presented in the study are included in the article/Supplementary Material, further inquiries can be directed to the corresponding authors.
