# Supplementary material for: Interaction of Colchicine-Site Ligands With the Blood Cell-Specific Isotype of β-Tubulin—Notable Affinity for Benzimidazoles
Source: Front Cell Dev Biol. 2022 May 31;10:884287. doi: 10.3389/fcell.2022.884287 (PMC9194530; doi:10.3389/fcell.2022.884287)
Supplement: Supplementary file 1 [file DataSheet1.PDF]

Supplementary Information File

Interaction of colchicine-site ligands with the blood cell-specific isotype of β-tubulin – Notable affinity for benzimidazoles

Running title: Chicken erythrocyte tubulin colchicine site

Felipe Montecinos [1 †], Maura Loew [2,3 †], Tak I. Chio [2,4], Susan L. Bane [2*] and Dan L. Sackett [1*]

1- Division of Basic and Translational Biophysics, *Eunice Kennedy Shriver* National Institute of Child Health and Human Development, National Institutes of Health, Bethesda, Maryland 20892, United States

2- Department of Chemistry, Binghamton University, State University of New York, Binghamton, NY, 13902, United States

3- Current address: Agilift LLC, Havertown, PA, United States

4- Current address: Department of Pathology and Laboratory Medicine, Institute on Aging and Center for Neurodegenerative Disease Research, University of Pennsylvania School of Medicine, Philadelphia, PA 19104

† These authors contributed equally to his work

*** Corresponding author:**

Dan L. Sackett, Ph.D.
NICHD / NIH
Bldg. 29B, Rm 1E10
9000 Rockville Pike
Bethesda, MD 20892 USA
Phone: 301-594-0358
sackettd@mail.nih.gov

Susan L. Bane, Ph.D
Department of Chemistry
25 Murray Hill Road
Binghamton University
State University of New York
Binghamton, NY 13902
Phone: 607-777-2927
sbane@binghamton.edu

Supplementary Table S1				
#	**Colchicine mimic - 3 rings**	**Source**	**Extinction Coefficient [a], M^{-1} cm^{-1}, (wavelength, nm) Solvent = DMSO unless noted**	**Reference**
1	Colchicine	Sigma Aldrich	$1.7x10^4$ (352)<br>PMEG buffer	352 (Chabin et al., 1990)
2	Colcemid	CIBA Geigy	$1.58x10^4$ (354)<br>PMEG buffer	354 (Pyles and Hastie, 1993)
3	Deacetylcolchicine (DAC)	Susan Bane	$1.40x10^4$ (352)<br>PMEG buffer	352 (Pyles and Hastie, 1993)
4	Desacetamidocolchicine (DAAC)	Susan Bane	$1.62x10^4$ (352)<br>PMEG buffer	352 (Pyles et al., 1992)
5	Isocolchicine	Susan Bane	$1.6x10^4$ (348)<br>H_2O	348 (Hastie et al., 1989)
6	Cornigerine	Ernest Hamel, NCI		
7	Steganacin	Ernest Hamel, NCI		
8	Allocolchicine	Susan Bane	$0.45x10^4$ (295)	295 (Sharma et al., 2010)
9	Allo methyl ketone (KAC)	Susan Bane	$1.55x10^4$ (295)<br>PME buffer	302 (Dumortier et al., 1997)
10	Allo ethyl ketone	Susan Bane	$1.5x10^4$ (302)	302
11	Thiocolchicine	Adipogen	$1.7x10^4$ (380)<br>PMEG buffer	380 (Chabin et al., 1990)
12	1,2-Didemethylcolchicine	Jan Wolff, NIDDK		
13	Podophyllotoxin	Sigma Aldrich	$0.37x10^4$ (290)<br>PMEG buffer	290 (Andreu and Timasheff, 1982)
14	Azatoxin	Yves Pommier, NCI		
15	Colchiceine	Susan Bane	40% DMSO / Mes buffer	
16	Trimethylcolchicinic acid	Susan Bane		
#	**Colchicine mimic - 2 rings**	**Source**	**Extinction Coefficient [a], M^{-1} cm^{-1}, (wavelength, nm) Solvent = DMSO unless noted**	**Reference**

17	AC (MTC)	Jan Wolff, NIDDK<br><br>Thomas J Fitzgerald, Florida A&M	$1.8x10^4$ (343)	343 (Menendez et al., 1989)
18	Combrestastatin (CS) A2	Ernest Hamel, NCI		
19	CS-A4 cis	Karl Werbovetz, Ohio State Univ.		
20	CS-A4 trans	Karl Werbovetz, Ohio State Univ.		
21	Dyhydro CS A4	Ernest Hamel, NCI		
22	Trimethoxyresveratrol (trans)<br>(3, 5, 4'-trimethoxystilbene)	Biomol		
23	Resveratrol			
24	MDL-27048	Susan Bane	$2.1x10^4$ (398)	398 (Peyrot et al., 1992)
**#**	**Other Structures**	**Source**	**Extinction Coefficient [a], M^{-1} cm^{-1}, (wavelength, nm) Solvent = DMSO unless noted**	**Reference**
25	Tubulazole C	Janssen Pharmaceuticals		
26	Tubulazole T	Janssen Pharmaceuticals		
27	Indanocine	Calbiochem		
28	T113242	Santa Cruz Biotechnology		
29	T138067	Tularik Pharmaceuticals		
30	ABT-751	Abbott Laboratories		
31	TN16	Biomol		
32	Tivantinib	Selleckchem		
33	Plinabulin	Selleckchem		
34	Lexibulin	Selleckchem		
35	Curvulin	Adipogen		
36	Indibulin (D248510)	Tocris		
37	Curvularin	Adipogen		
38	Dehydrocurvularin	Adipogen		
39	Tryprostatin	Santa Cruz Biotechnologies		
40	2-methoxyestradiol (2-ME)	Calbiochem		
41	Berberine	Sigma Aldrich	50% DMSO / H_2O	
42	D-64131	Tocris		

43	Ferulenol	Santa Cruz Biotechnologies		
**#**	**Benzimidazoles**	**Source**	**Extinction Coefficient [a], M^{-1} cm^{-1}, (wavelength, nm) Solvent = DMSO unless noted**	**Reference**
44	Nocodazole	Sigma Aldrich	$1.4x10^4$ (324)	(Sharma et al., 2010)
45	Mebendazole	Sigma Aldrich	$1.4x10^4$ (321)	
46	Thiabendazole	Sigma Aldrich		
47	Carbendazim	Sigma Aldrich	$0.74x10^4$	
48	Fenbendazole	Sigma Aldrich	$1.6x10^4$	
49	Flubendazole	LKT		
50	Albendazole	Sigma Aldrich	$1.2x10^4$	
51	Ricobendazole	LKT		
52	Oxibendazole	Sigma Aldrich	$1.3x10^4$	
53	Benomyl	Sigma Aldrich		

a. Solutions of compounds without extinction coefficients were prepared by weight.
b. PME and PMEG are the same as PM buffer (see main text), with additions of 0.5 mM EGTA (E), and 0.5 mM GTP (G).

Supplementary Figure S1

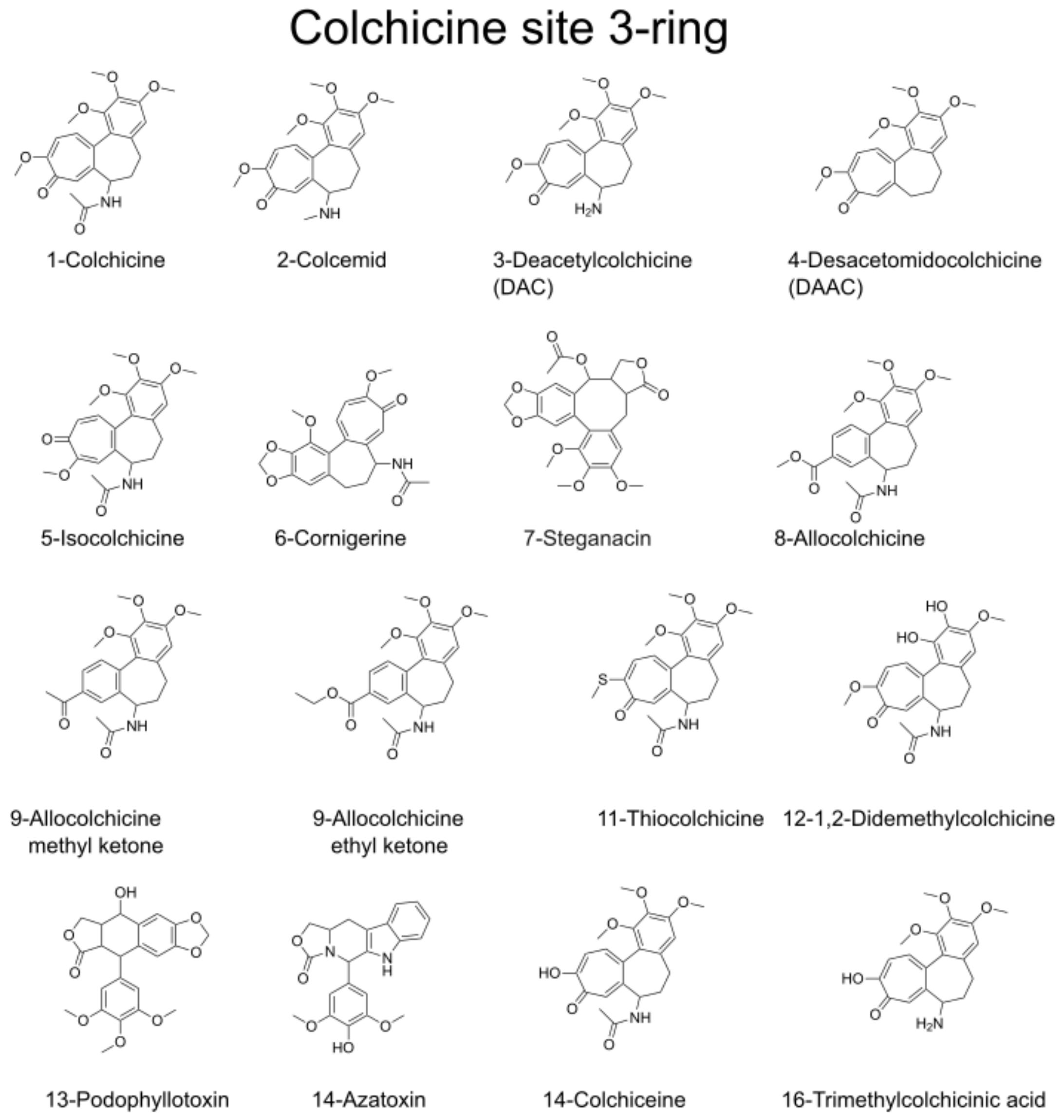

Supplementary Figure S1. Structures of drugs used in this study belonging to group 1: Colchicine site 3-ring. 1 = Colchicine, 2 = Colcemid, 3 = Deacetylcolchicine (DAC), 4 = Desacetamidocolchicine (DAAC), 5 = Isocolchicine, 6 = Cornigerine, 7 = Steganacin, 8 = Allocolchicine, 9 = Allocolchicine methyl ketone, 10 = Allocolchicine ethyl ketone, 11 = Thiocolchicine, 12 = 1,2-Didemethylcolchicine, 13 = Podophyllotoxin, 14 = Azatoxin, 15 = Colchiceine, 16 = Trimethylcolchicinic acid.

Supplementary Figure S2

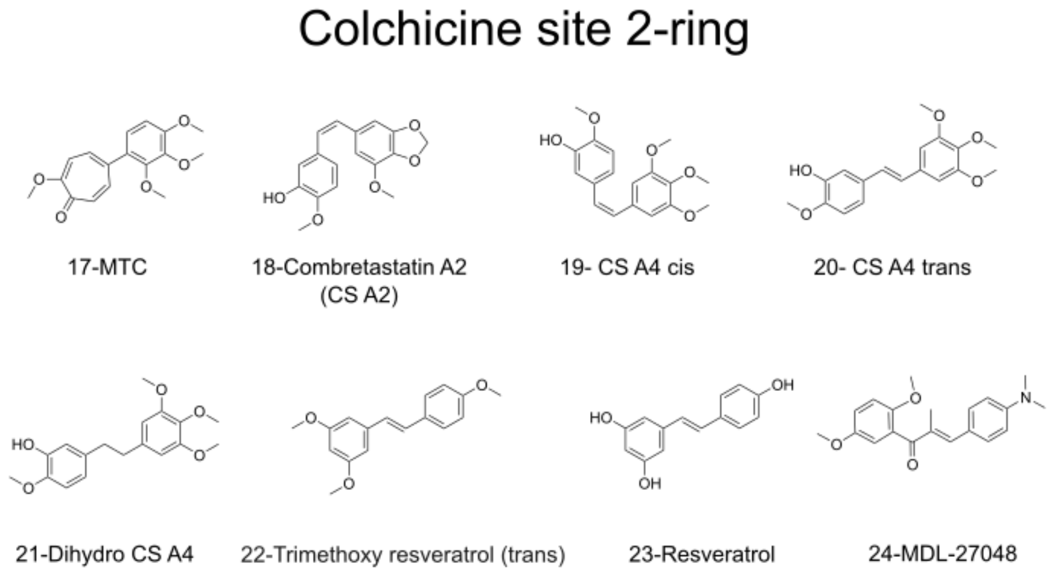

Supplementary Figure S2. Structures of drugs used in this study belonging to group 2: Colchicine site 2-ring. 17 = AC (MTC), 18 = Combrestastatin (CS) A2, 19 = CS-A4 cis, 20 = CS-A4 trans, 21 = Dihydro CS A4, 22 = Trimethoxy resveratrol (trans), 23 = Resveratrol, 24 = MDL-27048.

Supplementary Figure S3

Other structures

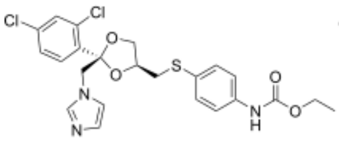

25-Tubulozole C

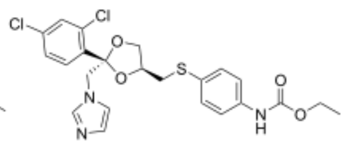

26-Tubulozole T

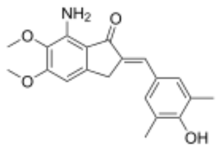

27-Indanocine

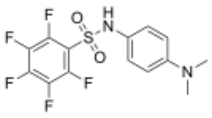

28-T113242

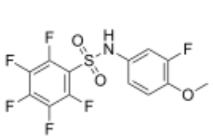

29-T138067

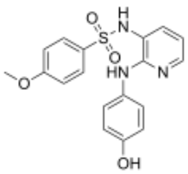

30-ABT-751

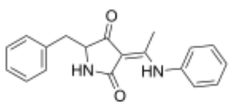

31-TN16

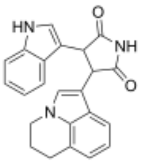

32-Tivantinib

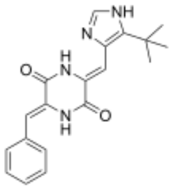

33-Plinabulin

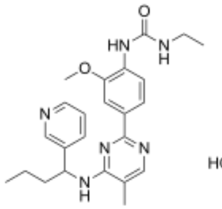

34-Lexibulin

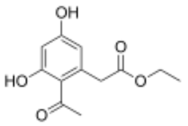

35-Curvulin

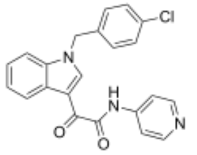

36-Indibulin

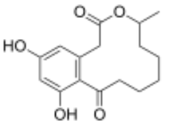

37-Curvularin

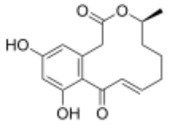

38-Dehydrocurvularin

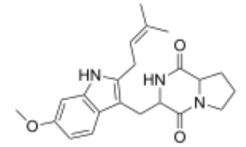

39-Tryprostatin

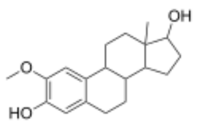

40-2-methoxyestradiol (2-ME)

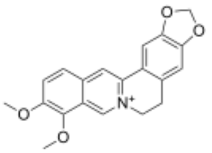

41-Berberine

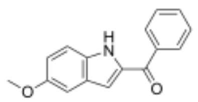

42-D-64131

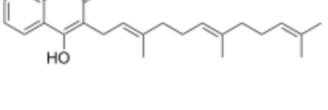

43-Ferulenol

Supplementary Figure S3. Structures of drugs used in this study belonging to group 3: Other structures. 25 = Tubulozole C, 26 = Tubulozole T, 27 = Indanocine, 28 = T113242, 29 = T138067, 30 = ABT-751, 31 = TN16, 32 = Tivantinib, 33 = Plinabulin, 34 = Lexibulin, 35 = Curvulin, 36 = Indibulin, 37 = Curvularin, 38 = Dehydrocurvularin, 39 = Tryprostatin, 40 = 2-methoxyestradiol (2-ME), 41 = Berberine, 42 = D-64131, 43 = Ferulenol.

Supplementary Figure S4

Benzimidazoles

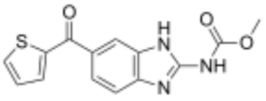

44-Nocodazole

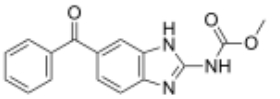

45-Mebendazole

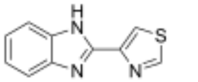

46-Thiabendazole

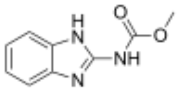

47-Carbendazim

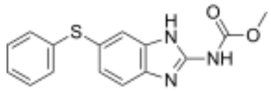

48-Fenbendazole

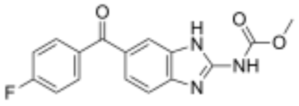

49-Flubendazole

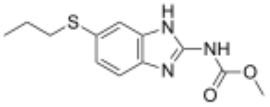

50-Albendazole

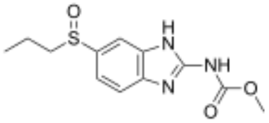

51-Ricobendazole

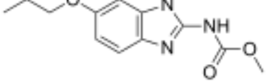

52-Oxibendazole

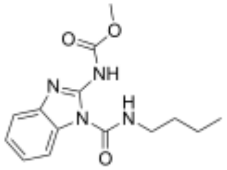

53-Benomyl

Supplementary Figure S4. Structures of drugs used in this study belonging to group 4: Benzimidazoles. 44 = Nocodazole, 45 = Mebendazole, 46 = Thiabendazole, 47 = Carbendazim, 48 = Fenbendazole, 49 = Flubendazole, 50 = Albendazole, 51 = Ricobendazole, 52 = Oxibendazole, 53 = Benomyl.

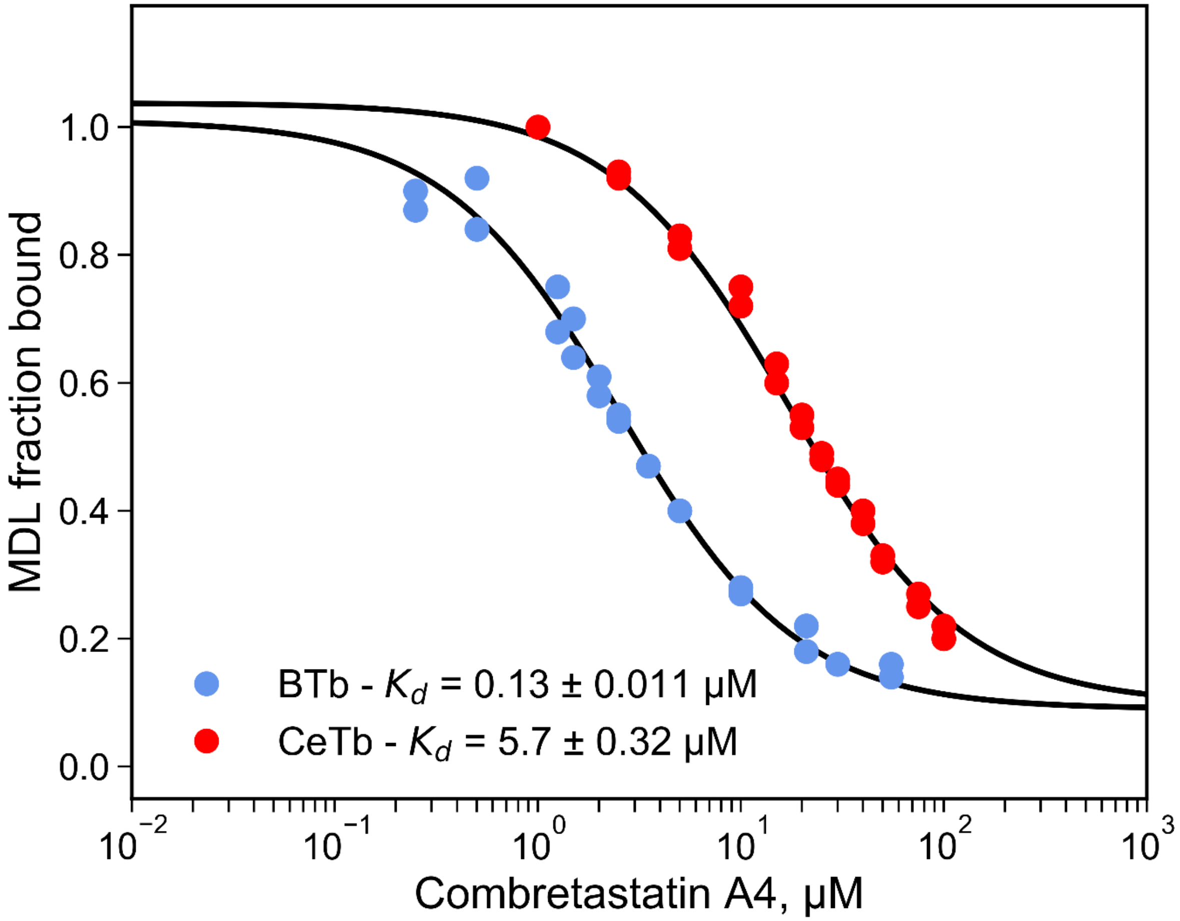

Supplementary Figure S5. Competition binding of combretastatin A4 cis with BTb and CeTb. Samples were prepared in duplicate containing 1 μM tubulin, 5 μM MDL-27048, varying concentrations of combretastatin A4, and 4% v/v DMSO in PMEG buffer. The fraction bound was determined from samples with no competing ligand (bound) and with saturating concentrations of competing ligand (free). The data were fit as described in the Materials and Methods section to obtain a dissociation constant of 5.7 ± 0.32 μM for the binding of combretastatin A4 to CeTb and 0.13 ± 0.011μM for BTb.

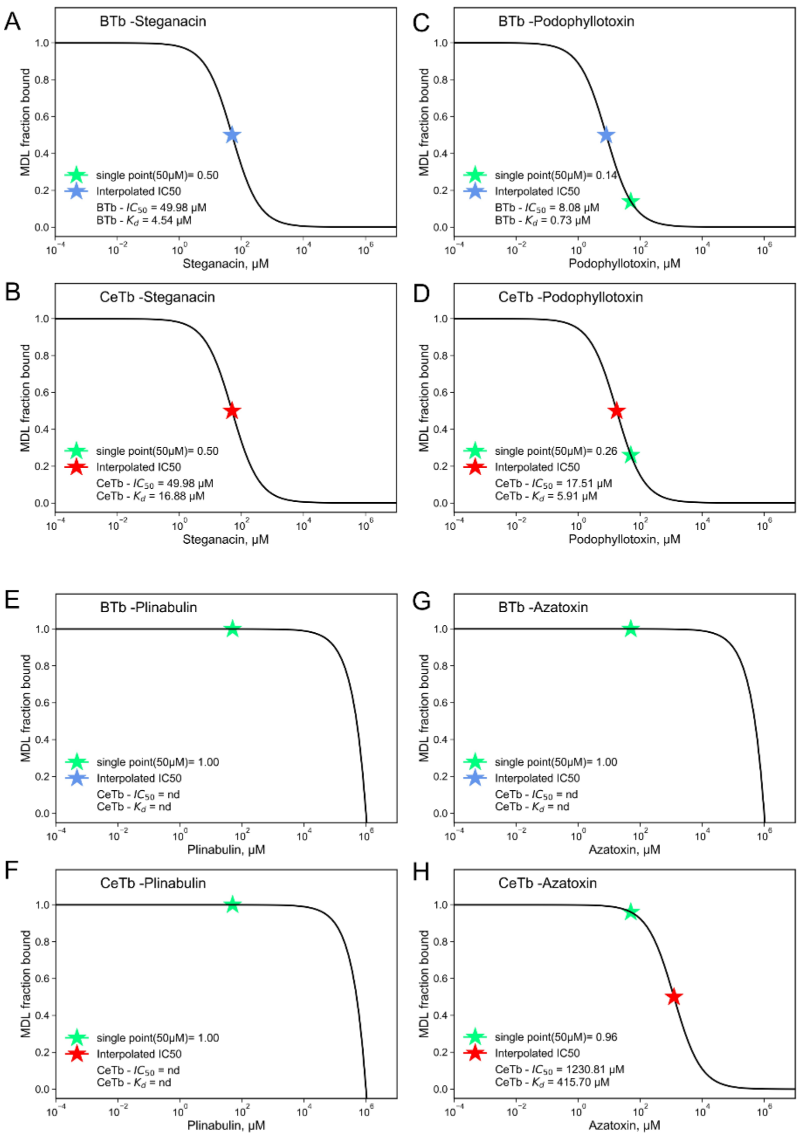

Supplementary Figure S6. Calculation of the apparent dissociation constant from single-point competition data. Single point data (green star in each plot) was plotted with the corresponding drug concentration (50 μM) and a two-state binding isotherm was fitted whenever possible, assuming a single binding site. Then, the midpoint of the best-fitted curve was obtained (blue star for BTb or red star for CeTb) which reflects the 50% inhibition of the binding model ($IC_{50\%}$). The fraction bound is inversely proportional to the % inhibition. The apparent dissociation constant was calculated using equation 4 and the best-fitted $IC_{50\%}$. Four examples are shown representing cases where BTb and CeTb gave the same % inhibition (A and B), similar but lower than 50% inhibition (C and D), both cases the procedure did not work due to low inhibition (<1%) (E and F), and cases where only one tubulin showed binding (G and H).

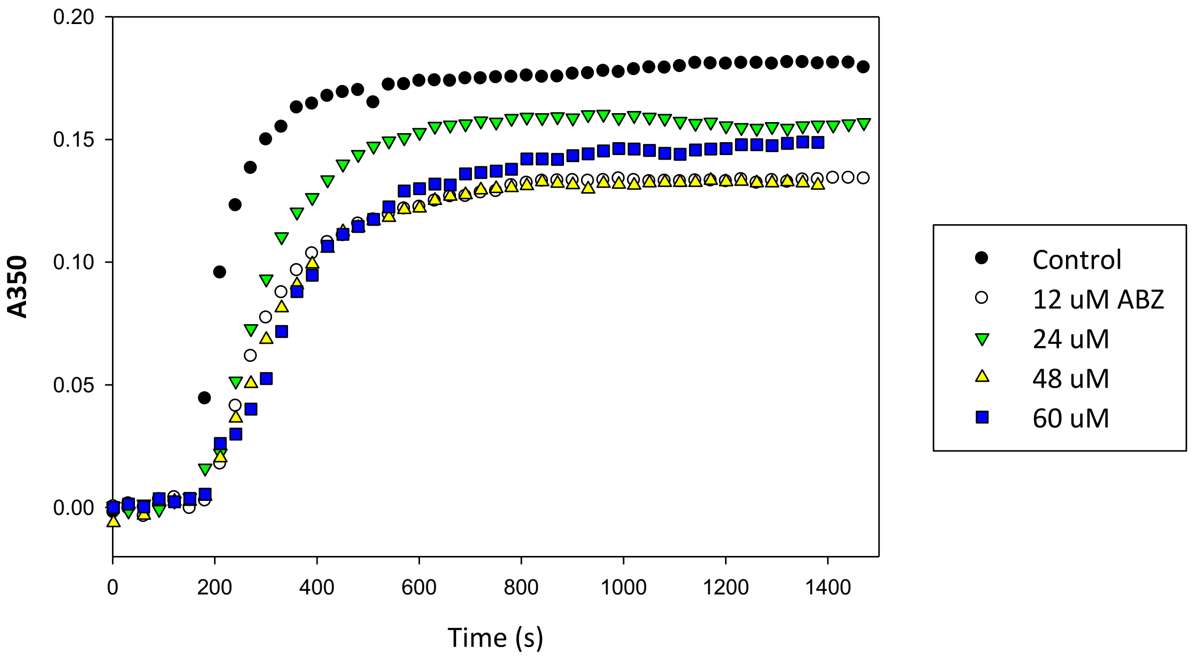

Supplementary Figure S7. BTb polymerization is not significantly inhibited by albendazole (ABZ). 15 µM bovine brain microtubule protein (tubulin plus microtubule-associated proteins, purified according to (Williams and Lee, 1982), in PME buffer was incubated with varying concentrations of albendazole for 40 min at room temperature. The samples were equilibrated to 37 °C in the cell holder of a UV-Vis spectrophotometer (HP8453, Hewlett Packard, Palo Alto, CA). A baseline was recorded before adding GTP to a concentration of 1 mM to induce microtubule assembly. All samples had 2% (v/v) DMSO. The extent of polymerization was determined by the increase of turbidity as measured by the apparent absorbance at 350 nm.

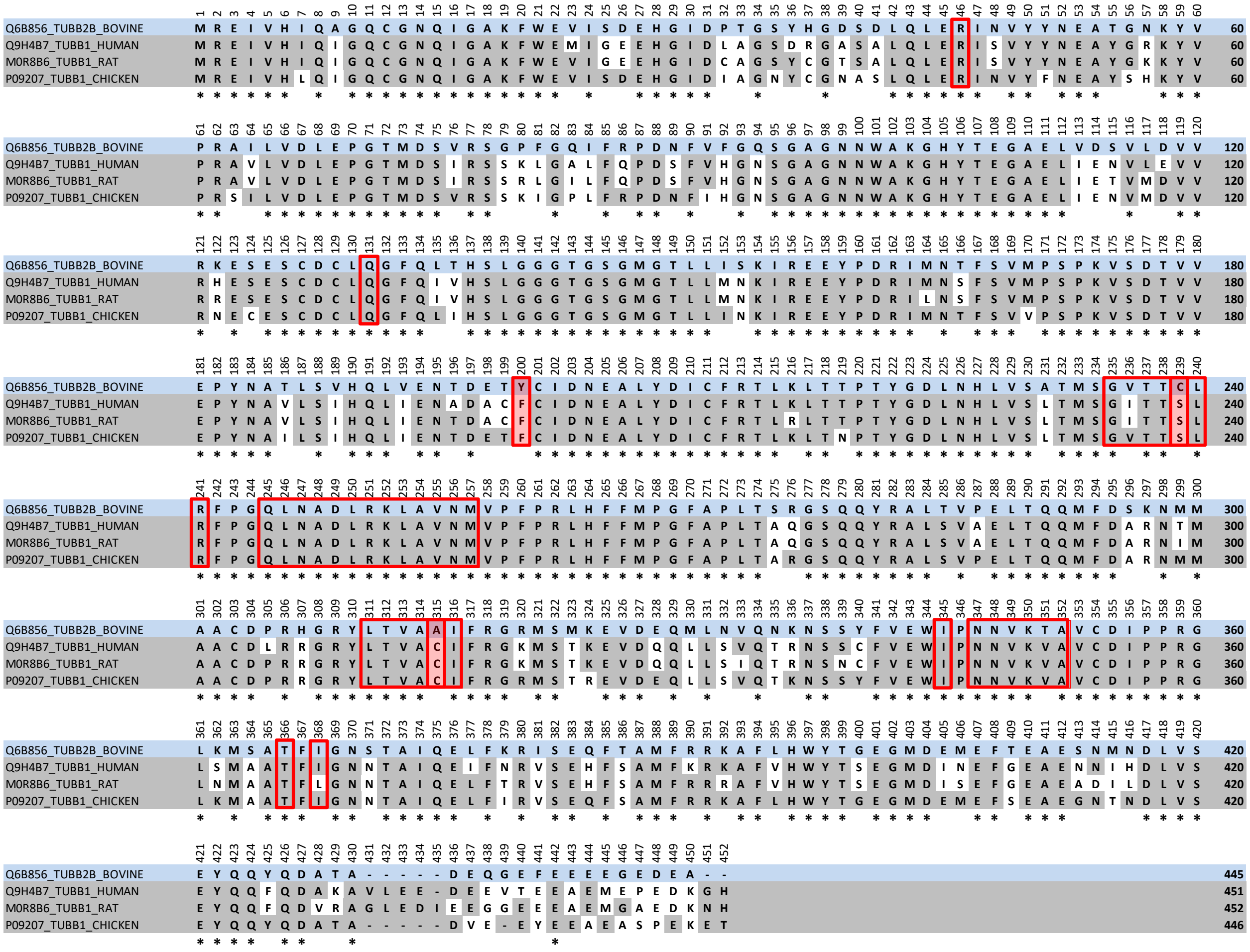
Q6B856_TUBB2B_BOVINE
Q9H4B7_TUBB1_HUMAN
M0R8B6_TUBB1_RAT
P09207_TUBB1_CHICKEN

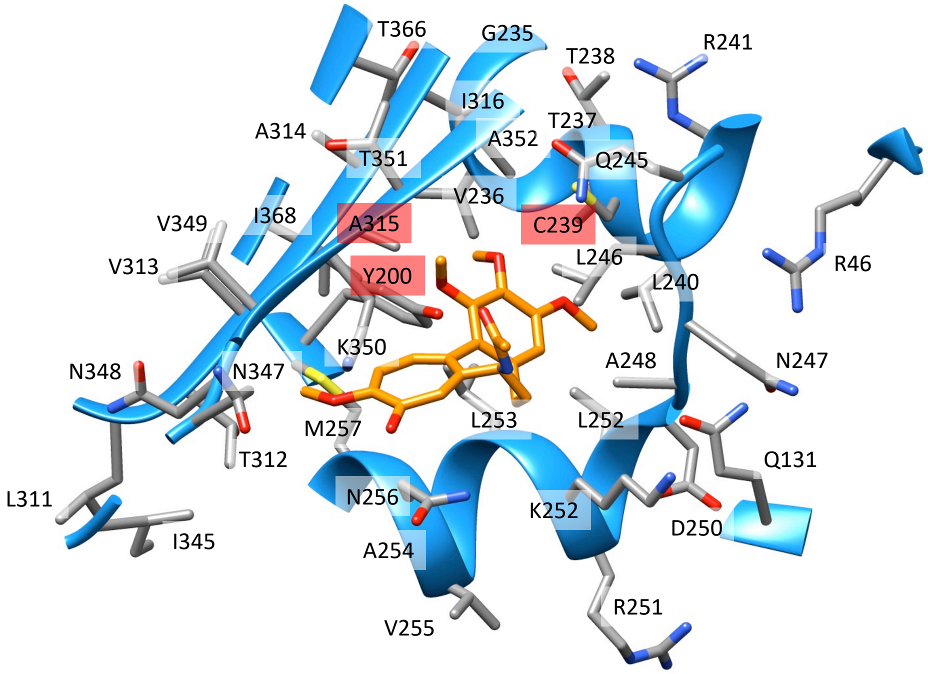
T366
G235
T238
R241
I316
T237
A314
A352
T351
Q245
V236
V349
I368
A315
C239
R46
V313
Y200
L246
L240
K350
N348
N347
A248
N247
M257
L253
L252
Q131
T312
L311
N256
K252
D250
I345
A254
R251
V255

Supplementary Figure S8. Sequence and structure of the β-tubulin colchicine binding site. Top panel, multisequence alignment of bovine brain β -tubulin (TUBB2B, light blue) and of chicken (rat and human) erythrocyte β -tubulin class VI (TUBB1, light gray). Residues different from TUBB2B are in white background. Stars at the bottom of the alignment indicate positions of residues identical to brain tubulin. Numbers above the sequences indicate the alignment position. Residues enclosed in red boxes are the 38 residues of β -tubulin within 6 Å of the bound colchicine (orange) in the crystal structure of bovine TUBB2B (4O2B.PDB). Bovine brain TUBB2B and chicken erythrocyte TUBB1 are 84.3% identical in total sequence. The residues substituted in the colchicine binding site of TUBB1 (Class VI) are highlighted in red: Y200F, C239S and A315C, meaning that the colchicine site residues of the two tubulins are 92% identical. Human TUBB1 and chicken TUBB1 differ in only 1 out of 38 residues (I236V : I = human),i.e., 97% identical. Bottom panel, tridimensional structure of the colchicine binding site in TUBB2B, ribbons are colored in blue and the side chains of the residues are in stick representation with oxygen and nitrogen atoms colored in red and blue, respectively. Residue numbers are in black, according to Uniprot Q6B856.

References

Andreu, J. M., and Timasheff, S. N. (1982). Conformational states of tubulin liganded to colchicine, tropolone methyl ether, and podophyllotoxin. *Biochemistry* 21, 6465–6476. doi:10.1021/bi00268a023.

Chabin, R. M., Feliciano, F., and Hastie, S. B. (1990). Effect of tubulin binding and self-association on the near-ultraviolet circular dichroic spectra of colchicine and analogues. *Biochemistry* 29, 1869–1875. doi:10.1021/BI00459A029.

Dumortier, C., Potenziano, J. L., Bane, S., and Engelborghs, Y. (1997). The mechanism of tubulin-colchicine recognition--a kinetic study of the binding of a bicyclic colchicine analogue with a minor modification of the A ring. *Eur. J. Biochem.* 249, 265–269. doi:10.1111/J.1432-1033.1997.T01-1-00265.X.

Hastie, S. B., Williams, R. C., Puett, D., and Macdonald, T. L. (1989). The binding of Isocolchicine to Tubulin. *J. Biol. Chem.* 264, 6682–6688. doi:10.1016/S0021-9258(18)83481-0.

Menendez, M., Laynez, J., Medrano, F. J., and Andreu, J. M. (1989). A thermodynamic study of the interaction of tubulin with colchicine site ligands. *J. Biol. Chem.* doi:10.1016/S0021-9258(19)84714-2.

Peyrot, V., Leynadier, D., Sarrazin, M., Briand, C., Menendez, M., Laynez, J., et al. (1992). Mechanism of binding of the new antimitotic drug MDL 27048 to the colchicine site of tubulin: Equilibrium studies. *Biochemistry* 31, 11125–11132. doi:10.1021/bi00160a024.

Pyles, E. A., and Hastie, S. B. (1993). Effect of the B Ring and the C-7 Substituent on the Kinetics of Colchicinoid-Tubulin Associations. *Biochemistry* 32, 2329–2336. doi:10.1021/bi00060a026.

Pyles, E. A., Hastie, S. B., Rava, R. P., and Harrison, G. R. (1992). Effect of B-Ring Substituents on Absorption and Circular Dichroic Spectra of Colchicine Analogs. *Biochemistry* 31, 2034–2039. doi:10.1021/bi00122a020.

Sharma, S., Poliks, B., Chiauzzi, C., Ravindra, R., Blanden, A. R., and Bane, S. (2010). Characterization of the colchicine binding site on avian tubulin isotype βVI. *Biochemistry* 49, 2932–2942. doi:10.1021/bi100159p.

Williams, R. C., and Lee, J. C. (1982). Preparation of Tubulin from Brain. *Methods Enzymol.* 85, 376–385. doi:10.1016/0076-6879(82)85038-6.
